# Correction: Nicholas-Haizelden et al. Bioprospecting the Skin Microbiome: Advances in Therapeutics and Personal Care Products. *Microorganisms* 2023, *11*, 1899

**DOI:** 10.3390/microorganisms14081784

**Published:** 2026-08-13

**Authors:** Keir Nicholas-Haizelden, Barry Murphy, Michael Hoptroff, Malcolm J. Horsburgh

**Affiliations:** 1 Institute of Infection, Veterinary and Ecological Sciences, University of Liverpool, Liverpool L69 3BX, UK; hlknicho@liverpool.ac.uk; 2 Unilever Research & Development, Port Sunlight, Wirral CH63 3JW, UK; barry.murphy@unilever.com (B.M.); michael.hoptroff@unilever.com (M.H.)

In the original publication [1], there was a mistake in Figure 1 as published. Apocrine glands have been incorrectly included on the scalp and forearm sections, which have been removed to accurate reflect skin structure at these sites. The corrected Figure 1 appears below. 

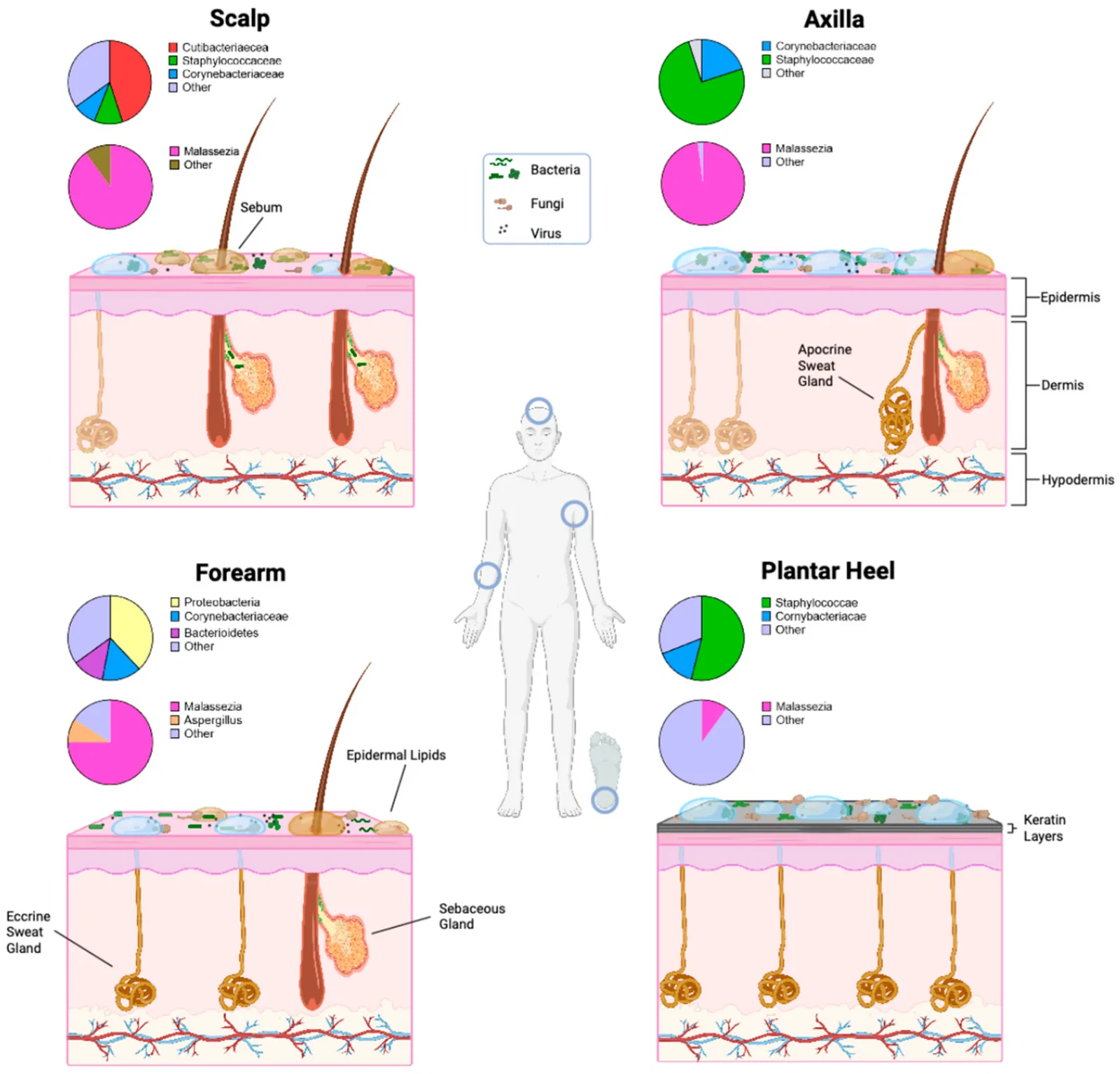



The authors state that the scientific conclusions are unaffected. This correction was approved by the Academic Editor. The original publication has also been updated.
